# Clinical Efficacy, Therapeutic Mechanisms, and Implementation Features of Cognitive Behavioral Therapy–Based Chatbots for Depression and Anxiety: Narrative Review

**DOI:** 10.2196/78340

**Published:** 2025-11-28

**Authors:** Chang-Ha Im, Minjung Woo

**Affiliations:** 1 School of Exercise and Sport Science, University of Ulsan, 93, Daehak-ro, Nam-gu, Ulsan, 44610, Republic of Korea, 82 52-259-2380

**Keywords:** CBT chatbot, artificial intelligence, depression, anxiety, digital psychotherapy

## Abstract

**Background:**

Cognitive behavioral therapy (CBT)–based chatbots, many of which incorporate artificial intelligence (AI) techniques, such as natural language processing and machine learning, are increasingly evaluated as scalable solutions for addressing mental health issues, such as depression and anxiety. These fully automated or minimally supported interventions offer novel pathways for psychological support, especially for individuals with limited access to traditional therapy.

**Objective:**

This narrative review synthesized evidence on the clinical efficacy, therapeutic mechanisms, and technological features of CBT-based chatbots designed to alleviate depressive and anxiety symptoms.

**Methods:**

Fourteen peer-reviewed studies published between January 2015 and March 2025 were identified through systematic searches and met predefined inclusion criteria. The studies were analyzed to extract information on intervention structure, therapeutic components, outcomes, and implementation characteristics.

**Results:**

Across the included studies, CBT-based chatbots consistently demonstrated short-term reductions in depressive symptoms, whereas findings for anxiety outcomes were mixed, with some studies reporting improvements and others showing nonsignificant or unreported effects. Moderate effect sizes were observed for depression. Reported therapeutic features included cognitive restructuring, behavioral activation, relaxation and mindfulness strategies, emotional support, self-monitoring and feedback, and therapeutic alliance. Technological characteristics such as real-time feedback and adaptive goal tracking were associated with enhanced engagement and adherence.

**Conclusions:**

CBT-based chatbots appear to be a promising and scalable modality for delivering psychological support, particularly for underserved populations. However, variability in study designs, heterogeneity of outcome reporting, and limited long-term evidence pose challenges for generalizability. Emerging evidence from generative AI chatbots (eg, Therabot and Limbic Care) highlights both opportunities and risks. Future work should examine long-term efficacy, adaptive personalization, cross-cultural adaptation, and rigorous ethical oversight.

## Introduction

Cognitive behavioral therapy (CBT) is a well-established and evidence-based psychological intervention for treating depression and anxiety. CBT alleviates emotional distress using structured techniques, such as cognitive restructuring and behavioral activation, which aim to modify negative thought patterns and maladaptive behavioral responses. Although face-to-face CBT has demonstrated efficacy across a wide range of mental health conditions [1,2], numerous individuals remain untreated because of barriers such as limited access to trained clinicians, high treatment costs, and social stigma [3,4].

In response to these barriers, conversational artificial intelligence (AI)–based chatbots have recently emerged as scalable tools for delivering CBT-informed support in digital contexts. A chatbot is a software agent that simulates interactive conversations with users through text or voice interfaces, functioning as a virtual coach or guide by delivering psychoeducational content and guiding therapeutic dialogue. Early prototypes such as ELIZA (developed at the Massachusetts Institute of Technology in the 1960s) [5] were limited to rule-based scripts and reflective mirroring.

CBT chatbots can be broadly categorized into 3 architectures, often described as an AI taxonomy of chatbots. Rule-based systems rely on preprogrammed scripts and decision trees, ensuring safety and consistency but limiting personalization. Hybrid models combine rule-based safeguards with natural language processing (NLP) and machine learning classifiers, enabling greater flexibility while maintaining clinical oversight (eg, Wysa and Youper). Generative models, powered by large language models, can produce open-ended responses and more naturalistic dialogue (eg, Therabot and Limbic Care) but require strict safeguards to mitigate risks of misinformation or hallucinated outputs. In this review, ‘chatbot’ is used as the primary term; ‘conversational agent’ is treated as a synonym.

Several CBT-based chatbot platforms, such as Woebot [6], Wysa [7], Tess [8], and Youper [9], have been specifically developed to alleviate symptoms of depression and anxiety. Unlike static mental health apps that merely present information, these chatbots actively engage users in therapeutic activities. For instance, users are guided to identify automatic negative thoughts and practice cognitive reframing strategies to challenge maladaptive cognitions [8,10]. These systems also recommend behavioral tasks, such as scheduling pleasant events or performing breathing exercises, within ongoing conversations. Some platforms incorporate advanced features, such as real-time mood tracking and personalized feedback in Youper [9], or crisis keyword detection in Wysa [7], enabling timely responses and adaptive guidance.

Despite the absence of a human therapist, several studies suggest that users may develop a sense of working alliance with these tools, as reported for Woebot [6] and Wysa [7]. Research indicates that chatbot interactions are often perceived as empathetic and emotionally supportive [7]. Techniques such as name personalization, affirming language, and nonjudgmental tone contribute to user engagement and perceived support. Although this alliance may be limited in depth compared to human-delivered therapy, studies indicate that it can be sufficient to enhance adherence and user satisfaction, as observed in studies that used Woebot [6] and Tess [8].

Preliminary findings from randomized controlled trials (RCTs) suggest that CBT chatbots can yield significant reductions in depression and anxiety over short-term interventions (typically 2‐8 weeks), particularly when compared to passive or information-only controls [6,8]. However, the mechanisms through which these outcomes are achieved—whether cognitive, behavioral, or relational—have not been systematically reviewed. Moreover, there remains a lack of synthesis regarding how technological design and implementation features contribute to therapeutic impact.

To address this gap, this narrative review synthesizes empirical findings from 14 studies on CBT-based mental health chatbots published between 2015 and 2025. It evaluates (1) the effectiveness of these interventions in reducing depressive and anxiety symptoms over short and long durations; (2) the therapeutic mechanisms, defined as CBT-consistent techniques, such as cognitive restructuring, behavioral activation, relaxation, mindfulness, emotional support, self-monitoring and feedback, and therapeutic alliance; and (3) the implementation features, defined as the design and deployment characteristics of chatbot platforms, including AI architecture (eg, rule based, hybrid, and generative), protocol structure (eg, modular vs open-ended), usability, acceptability, and user engagement. By integrating findings across clinical and technological domains, this review aims to inform the development of more effective, engaging, and ethically robust CBT-based mental health tools.

## Methods

### Narrative Review Approach

This narrative review aimed to synthesize empirical evidence on the clinical efficacy, therapeutic mechanisms, and implementation features of CBT-based mental health chatbots.

Given the field’s early stage and heterogeneity, we used a narrative synthesis. This approach allowed for theoretical integration and contextual interpretation across diverse interventions situated at the intersection of clinical psychology, AI, and digital health.

The narrative format enabled a broad, theory-informed synthesis, meaning that we not only summarized empirical outcomes but also interpreted them in light of established CBT theory and digital health implementation frameworks. This approach allowed us to integrate therapeutic mechanisms, user experiences, and platform characteristics across heterogeneous study designs.

### Search Strategy

A comprehensive literature search was conducted in March 2025 across 4 academic databases—PubMed, Scopus, PsycINFO, and Web of Science—using the following Boolean query: (chatbot OR “conversational agent” OR “dialogue system”) AND (“cognitive behavioral therapy” OR CBT) AND (depression OR anxiety OR “mental health”). Searches were applied to titles, abstracts, and keywords and were restricted to English-language, peer-reviewed articles published from January 1, 2015, to March 31, 2025.

The 2015 start date was selected because the first empirical evaluations of CBT-based chatbots appeared in 2015. Before 2015, chatbot-related publications were largely conceptual discussions, technical prototypes, or rule-based systems (eg, ELIZA) that did not constitute empirical clinical trials. In addition, we performed backward and forward citation chasing (manual screening of reference lists and Google Scholar citation tracking) to identify any studies missed by the database search.

### Inclusion and Exclusion Criteria

Articles were included if they met all the following criteria. First, the intervention used a predominantly automated conversational agent delivering CBT-based techniques without real-time input from a human therapist. For this review, *fully automated* was defined as interventions where the chatbot itself delivered the therapeutic content. Minimal human involvement (eg, technical support and automated reminders) was permitted. Hybrid models were included only if the human role was limited to nonclinical tasks, such as technical assistance or scheduling, but excluded if a human provided real-time therapeutic decision-making, coaching, or guidance. The second inclusion criterion was that the study reported quantitative outcomes related to depression, anxiety, or other closely related mental health indicators. Third, the study used an RCT, quasi-experimental design, or uncontrolled pilot trial. Fourth, the article was written in English and published in a peer-reviewed journal.

Articles were excluded if they met any of the following criteria: (1) the intervention addressed only depression or anxiety without incorporating a chatbot; (2) the study lacked a clearly articulated evidence-based rationale for the chatbot’s therapeutic framework; (3) the chatbot was unrelated to mental health or used therapeutic approaches not grounded in CBT (eg, mindfulness alone, psychoanalysis); and (4) the study was a literature review, meta-analysis, protocol, or theoretical paper without original intervention data.

### Study Selection

After removing duplicates, both authors independently screened the titles and abstracts to identify studies relevant to the review objectives. Full texts of potentially eligible articles were then independently assessed against the inclusion criteria. Disagreements were first discussed between the 2 reviewers; if consensus could not be reached, a third reviewer was consulted to adjudicate. This multistep procedure ensured methodological rigor and minimized subjective bias.

### Data Extraction and Synthesis

For each included study, data were extracted using a standardized coding template developed a priori in Microsoft Excel to enhance reproducibility. Two authors piloted the form on 2 studies to ensure consistency and then applied it across the full dataset. Extracted data included target population, sample size, chatbot name and platform, intervention duration, core therapeutic components, outcome measures, and key findings on depression and anxiety.

We also reviewed process-related indicators, such as user satisfaction, engagement metrics, and dropout rates. On the basis of thematic coding of intervention descriptions, we identified and synthesized core therapeutic mechanisms and representative dialogue examples from the chatbots. This inductive thematic approach allowed key CBT strategies to emerge from the data.

Finally, we conducted a comparative analysis of technological and clinical features of the included chatbot platforms, focusing on AI architecture, protocol structure, usability, and user engagement outcomes. All coding and synthesis procedures were independently conducted and jointly reviewed by both authors to ensure rigor and conceptual alignment.

### Quality Assessment

The methodological quality of the included studies was assessed using the Cochrane Risk of Bias 2.0 (RoB 2.0) tool for RCTs and the Appraisal Tool for Cross-Sectional Studies (AXIS) for observational studies. Two reviewers (ie, the 2 authors) independently appraised each study, and disagreements were resolved through discussion. The quality ratings were incorporated into the synthesis to contextualize study findings and highlight areas where stronger evidence is needed.

## Results

### Study Selection

This narrative review was conducted to synthesize evidence on the effects of CBT-based chatbots for depression and anxiety. The literature search covered peer-reviewed journal articles published between March 1, 2015, and March 31, 2025, across 4 electronic databases: PubMed, Scopus, Web of Science, and PsycINFO.

The search initially yielded a total of 347 records (PubMed=71, Scopus=174, Web of Science=86, and PsycINFO=16). After removing 83 duplicates using a reference management tool, 264 records remained for title and abstract screening. At this stage, 211 records were excluded for reasons such as being unrelated to the research topic, not involving CBT-based interventions, or not applying chatbot technologies.

The full texts of the remaining 53 articles were then reviewed for eligibility. A total of 39 articles were excluded at this stage for the following reasons: interventions not based on CBT (n=14); nonchatbot or insufficient automation (n=10); no depression or anxiety outcomes reported (n=9); or reviews, meta-analyses, or protocols without original empirical data (n=6).

Ultimately, 14 studies met all inclusion criteria and were included in the narrative review for qualitative synthesis.

### Overview of Included Studies

A total of 14 studies met the inclusion criteria and were synthesized in the final review (Table 1). Of these, 10 were RCTs, 1 was a single-group pre-post trial, and 3 were observational or naturalistic studies. The studies were conducted across diverse countries, including the United States, the United Kingdom, China, Poland, Sweden, Canada, and South Korea, reflecting a broad international scope.

Sample characteristics varied considerably. Several RCTs recruited college or university students, including 70 students in the United States [6], 75 in the United Kingdom [8], 148 in China [10], and 28 in Sweden [11]. Other RCTs targeted specific populations, such as 192 postpartum women in the United States [12], 68 adults with chronic medical conditions such as arthritis or diabetes in Canada [13], and 98 adults with insomnia in South Korea [14]. Two trials were conducted in subclinical or community samples, including 81 young adults in Poland [15] and 103 young adults in the United States [16]. The most recent RCT tested a generative AI chatbot in 210 adults with major depression, generalized anxiety disorder, or eating disorder risk in the United States [17].

Observational and pre-post studies included large-scale naturalistic samples. Mehta et al [9] analyzed data from 4517 paying users of the Youper app (United States/global), whereas Inkster et al [7] examined 129 Wysa users worldwide. Prochaska et al [18] tested Woebot-SUD in 101 US adults with substance use problems, and Habicht et al [19] evaluated the Limbic Care tool in 244 patients undergoing group CBT within the UK NHS Talking Therapies program.

Intervention delivery formats also varied. Most chatbots were mobile app based (eg, Woebot, Wysa, XiaoE, Fido, Youper, Limbic Care), whereas Tess and CBT-txt were delivered via SMS text messaging or text message. The duration of chatbot use ranged from 1 to 8 weeks, with some trials incorporating longer follow-ups, such as 3 to 4 months [14,16] or 8 weeks [17].

Core therapeutic elements were consistent with CBT. Across the 14 studies, commonly implemented techniques included psychoeducation, cognitive restructuring, mood monitoring, behavioral activation, relaxation training, and emotion regulation strategies. Depression and anxiety were the primary outcomes, most frequently assessed with validated instruments, such as the Patient Health Questionnaire-9 (PHQ-9) and the Generalized Anxiety Disorder-7 (GAD-7).

Collectively, these studies provide evidence that CBT-based chatbots are being tested across diverse populations, countries, and delivery formats, demonstrating both scalability and adaptability in digital mental health care.

**Table 1.** Summary of included studies (all studies in this review) examining the clinical efficacy, therapeutic components, and outcomes of AI^a^-based CBT^b^ chatbots. Effect sizes and 95% CIs are listed when reported in the original studies. Where these were not reported, NR^c^ is indicated.

Study (citation)	Chatbot platform	Design type	Population and duration	Primary CBT techniques	Outcome measures	Main findings
Fitzpatrick et al [6]	Woebot	RCT^d^ (2 weeks)	70 US college students	Psychoeducation, cognitive restructuring, behavioral activation, mood tracking	PHQ-9^e^, GAD-7^f^, PANAS^g^	↓ Depression versus control (*d*=0.44; CI NR); anxiety NS^h^; attrition lower versus control; alliance NR; safety NR
Fulmer et al [8]	Tess (SMS chatbot)	RCT (2‐4 weeks, 3-arm)	75 US university students	Cognitive reframing, coping skills, psychoeducation	PHQ-9, GAD-7, PANAS	↓ Depression and anxiety versus eBook; attrition 0%‐4% (ES^i^ NR); attrition ~0% in chatbot group; alliance NR; safety NR
He et al [10]	XiaoE (China)	RCT (1 week+1-month FU^j^, 3-arm)	148 Chinese college students	Cognitive restructuring, mood monitoring, supportive dialogue	PHQ-9, GAD-7, WAQ^k^, usability scales	XiaoE >eBook and generic chatbot on depression; strong alliance (ES NR); higher acceptability & alliance versus controls; safety NR
Karkosz et al [15]	Fido (Poland)	RCT (2 weeks+1-month FU)	81 subclinical young adults	Psychoeducation, cognitive reframing, behavioral activation	CESD-R^l^, PHQ-9, PSWQ^m^, STAI^n^	↓ Depression/anxiety; sustained at 1-month FU (ES NR); engagement NR; alliance NR; safety NR
MacNeill et al [13]	Wysa	RCT (4 weeks)	68 Canadian adults with arthritis/diabetes	Cognitive restructuring, mindfulness, relaxation	PHQ-9, GAD-7, PSS-10^o^	↓ Depression/anxiety versus control (ES NR); engagement NR; alliance NR; safety NR
Suharwardy et al [12]	Woebot (perinatal)	RCT (6 weeks)	192 US postpartum women	Psychoeducation, cognitive restructuring, behavioral activation	PHQ-9, EPDS^p^, GAD-7	PHQ-9 improved versus usual care; EPDS/GAD-7 NS (ES NR); completion 74%; satisfaction 91%; alliance NR; safety NR
Shin et al [14]	Somzz (CBT-I^q^ app)	RCT (6 weeks+4-month FU, single blind)	98 South Korean adults with insomnia	CBT-I: stimulus control, sleep restriction, relaxation, cognitive therapy	ISI^r^ (primary), ESS^s^, DBAS^t^, PHQ-9, GAD-7	Large ISI improvement (*d*>1.0; CI NR); depression/anxiety NS; dropout ~12%; alliance NR; safety NR
Mason et al [16]	CBT-txt (SMS program)	RCT (8 weeks+3-month FU)	103 US young adults with depression	Behavioral activation, cognitive restructuring (via SMS text messaging)	BDI-II^u^, PHQ-9, PTQ^v^, CDS^w^	↓ Depression (*d*≈0.76; CI NR); ↓ rumination; engagement NR (reported strong adherence); alliance NR; safety NR
Heinz et al [17]	Therabot (Generative AI)	RCT (4 weeks+8-week FU)	210 US adults with MDD^x^, GAD, eating disorder risk	Psychoeducation, cognitive restructuring, behavioral activation, emotion regulation	PHQ-9, GAD-Q-IV, weight concerns scale, engagement	↓ Depression 51% (*d*≈0.85; CI NR); ↓ anxiety 31% (*d*≈0.84; CI NR); ↓ eating concerns 19% (*d*≈0.82; CI NR); strong alliance WAI-SR^y^≈3.6; engagement average 260 messages, 24 days, 6.2 hours; safety included crisis-detection protocol
Ly et al [11]	Shim (Sweden)	Pilot RCT (2 weeks)	28 Swedish community adults	Positive psychology+CBT (reframing, activation)	Flourishing scale, SWLS^z^, PSS-10, depression/anxiety (secondary)	↑ Flourishing/life satisfaction; modest ↓ depression/anxiety (ES NR); completion 100%; alliance NR; safety NR
Prochaska et al [18]	Woebot-SUD	Pre-post (8 weeks)	101 US adults with substance use problems	Cognitive restructuring, coping skills, motivational support, behavioral activation	AUDIT-C^aa^, DAST-10^ab^; PHQ-8, GAD-7 (secondary)	↓ Substance use; ↓ depression/anxiety; high attrition (~50%) (ES NR); alliance NR; safety NR
Inkster et al [7]	Wysa	Observational (≥2 weeks)	129 global app users	Cognitive restructuring, mindfulness, behavioral activation	PHQ-9	High-use group had greater ↓ depression; dose–response observed (ES NR); retention 33% at 2 weeks; 20% used ≥2 times per week; alliance NR; safety NR
Mehta et al [9]	Youper	Observational longitudinal (4 weeks)	4517 paying app users (US/global)	Cognitive restructuring, emotion regulation, mood monitoring	PHQ-9 (modified), GAD-7	↓ Depression/anxiety; dose–response confirmed (ES NR); large naturalistic cohort; alliance NR; safety NR
Habicht et al [19]	Limbic Care (UK NHS)	Observational cohort (6-session group CBT, 8-10 weeks)	244 patients in NHS Talking Therapies (England)	Between-session CBT support: psychoeducation, reframing, coping practice	PHQ-9, GAD-7, NHS^ac^ recovery metrics, attendance/dropout	↑ Recovery +25pp^ad^ (OR^ae^≈2.81; CI NR); ↑ reliable improvement;+21 pp (OR≈2.21; CI NR); ↓ dropout −23% (OR≈0.32; CI NR); ↑ attendance +42%; alliance NR; safety NR

a AI: artificial intelligence.

b CBT: cognitive behavioral therapy.

c NR: not reported.

d RCT: randomized controlled trial.

e PHQ-9: Patient Health Questionnaire-9.

f GAD-7: Generalized Anxiety Disorder-7.

g PANAS: Positive and Negative Affect Schedule.

h NS: not significant.

i ES: effect size.

j FU: follow-up.

k WAQ: Working Alliance Questionnaire.

l CESD-R: Center for Epidemiologic Studies Depression Scale–Revised.

m PSWQ: Penn State Worry Questionnaire.

n STAI: State–Trait Anxiety Inventory.

o PSS-10: Perceived Stress Scale.

p EPDS: Edinburgh Postnatal Depression Scale.

q CBT-I: cognitive behavioral therapy for insomnia.

r ISI: Insomnia Severity Index.

s ESS: Epworth Sleepiness Scale.

t DBAS: Dysfunctional Beliefs and Attitudes About Sleep Scale.

u BDI-II: Beck Depression Inventory-II.

v PTQ: Perseverative Thinking Questionnaire.

w CDS: Cognitive Distortions Scale.

x MDD: major depressive disorder.

y WAI-SR: Working Alliance Inventory–Short Revised.

z SWLS: Satisfaction With Life Scale.

aa AUDIT-C: Alcohol Use Disorders Identification Test–Consumption.

ab DAST-10: Drug Abuse Screening Test-10.

ac NHS: National Health Service.

ad pp: percentage points.

ae OR: odds ratio.

### Quality Assessment

The quality appraisal of included studies revealed variability in methodological rigor. Among the RCTs, 2 studies [10,14] were judged to have an overall low risk of bias, supported by robust randomization procedures, preregistration, and appropriate handling of missing data. In contrast, the majority of RCTs were rated as having some concerns, largely attributable to open-label designs, limited blinding, or moderate levels of attrition [6,8,11-13,15-17].

For the nonrandomized and observational studies, the overall judgment was moderate to high risk of bias. Recurring limitations included self-selected or convenience sampling frames, absence of randomization and control groups, high attrition, and reliance on self-reported outcomes [7,9,18,19]. Despite these methodological weaknesses, these studies provided valuable evidence regarding feasibility, acceptability, and real-world engagement with CBT-based chatbots.

**Figure 1.**
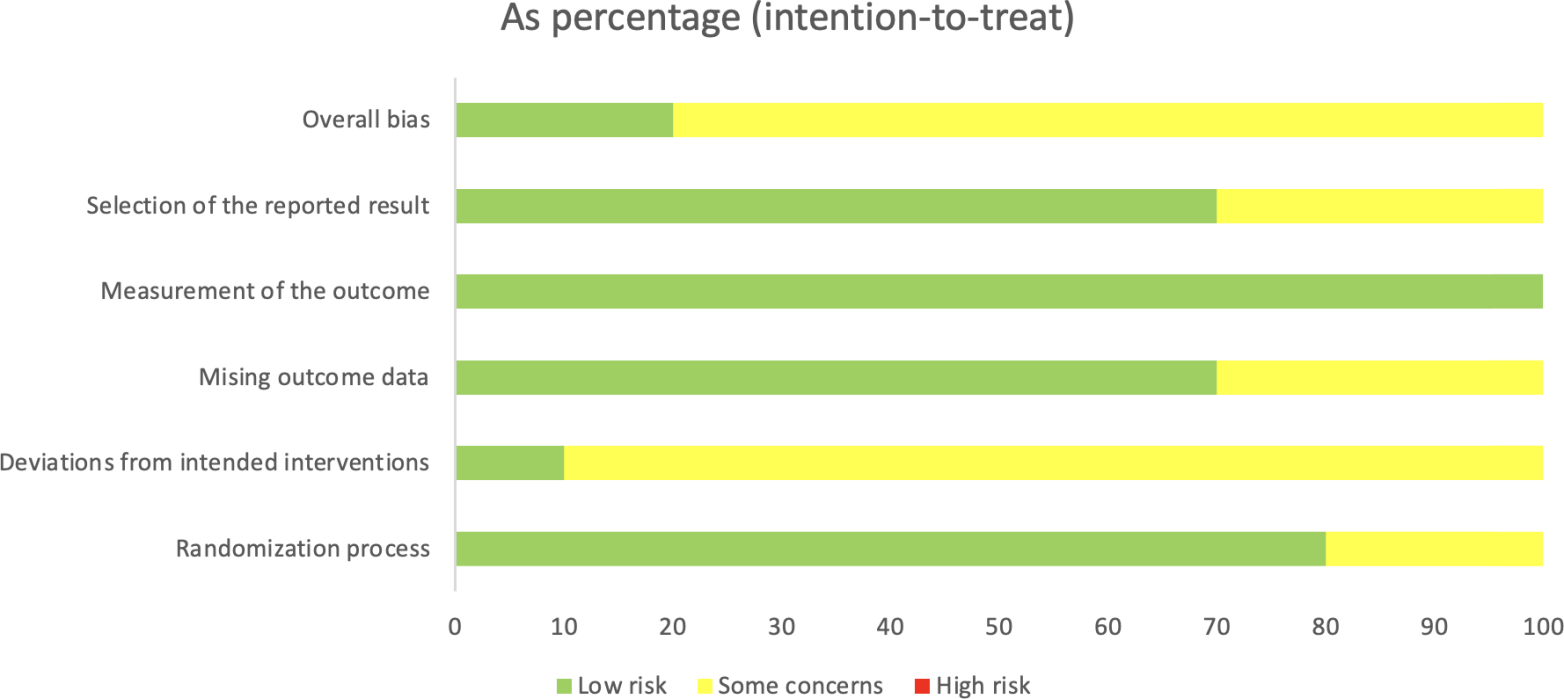
Risk of bias summary.

A detailed summary of risk of bias judgments across domains for each study is presented in Figure 1 and Table 2.

**Table 2.** Quality appraisal of observational studies using the AXIS^a^ tool.

Study (Chatbot)	Total score (out of 20)	Overall risk	Notes
Prochaska et al [18] (Woebot-SUD)	11/20	Moderate–high	Strength: clear aims, validated tools, ethics approval; weakness: convenience sampling, high attrition (~50%), no control group
Inkster et al [7] (Wysa)	10/20	Moderate–high	Strength: validated PHQ-9^b^, transparent reporting; weakness: self-selected users, inclusion only of ≥2 datapoints, high attrition, limited ethics reporting
Mehta et al [9] (Youper)	13/20	Moderate–high	Strength: very large N (4,517), multilevel models, validated GAD-7^c^; weakness: modified PHQ-9, female-dominant, self-selected paying users
Habicht et al [19] (Limbic Care)	15/20	Moderate–high	Strength: NHS^d^ service setting, routine PHQ-9/GAD-7, regression with covariate adjustment, ethics via NHS audit; weakness: self-selection of tool use, nonrandomized design

a AXIS: Appraisal Tool for Cross-Sectional Studies.

b PHQ-9: Patient Health Questionnaire-9.

c GAD-7: Generalized Anxiety Disorder-7.

d NHS: National Health Service.

### User Engagement and Acceptability

To provide context for interpreting efficacy, engagement outcomes are presented first. Engagement was defined variably across studies as program completion, threshold number of sessions, weekly active use/retention, or service-level attendance and dropout.

Completion rates in RCTs generally ranged from ~50% to 85%. For example, the postpartum Woebot trial [12] reported a 74% completion rate and 91% satisfaction, whereas Tess [8] reported virtually no attrition over 2 to 4 weeks. In contrast, Woebot-SUD [18] showed high attrition (~50%). Observational studies demonstrated dose–response relationships, such as Wysa users engaging ≥2 times per week experiencing >5-point reductions in PHQ-9 [7] and Youper users (n=4517) achieving an average 3.6-point PHQ-9 reduction within 2 weeks with higher usage intensity [9]. In a service context, the NHS evaluation of Limbic Care [19] found attendance increased by 42% and dropout decreased by 23% compared to usual care.

Detailed engagement definitions and metrics for each study are summarized in the *Main findings* column of Table 1, with “NR” indicated when not reported.

### Therapeutic Alliance

Therapeutic alliance was not uniformly assessed across studies. When measured, standardized or study-specific instruments were used. For example, Heinz et al [17] applied the Working Alliance Inventory–Short Revised and reported moderate alliance scores (mean ≈3.6). He et al [10] used study-specific items and found significantly higher alliance for XiaoE compared to an e-book control. Most other studies, including the postpartum Woebot trial [12], did not directly assess alliance but instead reported satisfaction or usability.

For transparency, alliance measurements are indicated in the *Main findings* column of Table 1, with “NR” used when not reported.

### Safety and Adverse Events

No study reported any serious adverse events directly attributable to chatbot use. Several platforms incorporated basic safety protocols, such as crisis keyword detection (eg, suicidal ideation) and automated referral messages to emergency resources. However, adverse event reporting was inconsistent and often not provided and is therefore indicated as NR. These details are summarized in the *Main findings* column of Table 1.

### Efficacy of CBT Chatbots for Depression and Anxiety

To ensure consistency across studies, efficacy outcomes are reported in a standardized manner: effect sizes with 95% CIs are presented when available; when not reported in the original study, point reductions on validated measures (eg, PHQ-9 and GAD-7) are included; if neither were available, findings are described narratively. This convention is reflected in Table 1, where studies with unreported data are indicated as NR.

Across the 14 included studies, CBT-based chatbots consistently demonstrated short-term reductions in depressive symptoms, whereas findings for anxiety symptoms were mixed: some trials reported improvements, whereas others found no significant effects or did not report anxiety outcomes. Table 1 provides an overview of each study’s sample, intervention, and outcomes. In the first RCT of a mental health chatbot, college students who interacted with Woebot for 2 weeks showed a greater decrease in depression (PHQ-9 scores) compared to an information-only control group [6]. Woebot delivered CBT strategies, including psychoeducation, cognitive restructuring, and behavioral activation. The between-group effect size for depression was moderate (*d*≈0.44), whereas anxiety symptoms did not differ significantly between groups. Similarly, Fulmer et al [8] found that students using the SMS text messaging–based chatbot Tess for 2 to 4 weeks reported significantly lower depression and anxiety levels postintervention than those using a psychoeducational e-book. The observed effect size for depression in that study was moderate-to-large (*d*=0.68), and no attrition was reported in the chatbot group.

Subsequent RCTs supported these findings. A 3-arm trial in China demonstrated that XiaoE, a CBT chatbot, produced significantly greater reductions in depressive symptoms compared to both a minimal control (e-book) and a nontherapeutic chatbot control, with a between-group effect size of *d*≈0.5 [10]. Users also reported higher therapeutic alliance and acceptability than those in the control groups. In Poland, Karkosz et al [15] evaluated the Polish-language chatbot Fido, which yielded significant reductions in both PHQ-9 and GAD-7 scores among subclinical young adults.

More recent RCTs extended this evidence. Shin et al [14] tested Somzz, a CBT for insomnia (CBT-I) chatbot, among Korean adults with insomnia and found large improvements in insomnia severity index (ISI, *d*>1.0) with modest or nonsignificant changes in depression and anxiety. Mason et al [16] reported that symptom improvements from an 8-week SMS text messaging–based CBT chatbot (CBT-TXT) were sustained at 3-month follow-up, whereas MacNeill et al [13] found that initial gains in adults with chronic conditions were not maintained without ongoing support.

Most recently, Heinz et al [17] conducted the first RCT of a generative AI therapy chatbot, Therabot. In this 4-week trial with 210 US adults presenting with depression, anxiety, or eating concerns, Therabot users showed significantly greater symptom improvements compared to a waitlist control group. The effect sizes were large for depression (*d*≈0.85), anxiety (*d*≈0.84), and eating concerns (*d*≈0.82), demonstrating the potential of generative AI to achieve clinical benefits comparable to traditional CBT.

Beyond controlled trials, real-world observational studies confirmed the feasibility of CBT chatbot use. In a large naturalistic sample (n>4500), Mehta et al [9] reported that Youper users experienced an average 3.6-point reduction in PHQ-9 scores within the first 2 weeks. Similarly, Inkster et al [7] found that Wysa users who engaged with the chatbot at least twice per week over 2 weeks reported PHQ-9 reductions of more than 5 points, suggesting a dose–response relationship between chatbot engagement and symptom improvement.

In the UK NHS Talking Therapies program, Habicht et al [19] evaluated Limbic Care, a generative AI (GenAI)–enabled support tool integrated with group CBT. In this multisite observational cohort (n=244), patients who used the AI tool demonstrated a 25-percentage-point higher recovery rate, 21-point higher reliable improvement, and 23% lower dropout compared to those receiving standard care alone.

In summary, CBT-based chatbots appear to offer meaningful short-term relief for depressive symptoms, whereas the evidence for anxiety outcomes remains mixed, with some studies reporting benefits but others showing nonsignificant or unreported effects. Evidence from both RCTs and real-world implementations supports their feasibility and acceptability. Emerging evidence also highlights the potential of generative AI chatbots (eg, Therabot and Limbic Care) to achieve substantial clinical effects and improve engagement, although further research is needed to confirm long-term safety and efficacy.

### Therapeutic Mechanisms and Implementation Characteristics of CBT Chatbots

Beyond symptom outcomes, included studies revealed 6 consistent therapeutic mechanisms used by CBT chatbots, as detailed in Table 3. These mechanisms align with the foundational components of face-to-face CBT and have been adapted for digital delivery through structured conversation modules and affect-sensitive prompts.

**Table 3.** Therapeutic mechanisms implemented in cognitive behavioral therapy–based chatbots, corresponding psychological effects, representative chatbot platforms, and illustrative examples of user-chatbot dialogue scripts.

Therapeutic mechanism	Main effect	Applied chatbot	Example of specific conversation script	Study
Cognitive restructuring	Depression; anxiety reduction; and decrease in negative thinking and cognitive distortions	Woebot, XiaoE, Youper, Fido, Therabot	*Woebot: Was there a hard time today?* *User: I made a mistake at the meeting and thought everyone believed I was incompetent.* *Woebot: Is there evidence that supports that idea is true?* *User: Nobody said that.* *Woebot: Can you interpret this situation from a different perspective?* *User: I guess one mistake doesn’t mean I’m incompetent.*	[6,9,10,12,15,17,18]
Behavioral activation	Increased activity and improved mood; reduction in depression and insomnia	Woebot, Wysa, Somzz, XiaoE, Youper, Therabot, Shim, CBT-TXT, Fido	*Wysa: Let’s set a small activity that can make you feel better today.* *User: I’m going to walk every morning.* *Wysa: It’s a good choice! Let’s decide what time you want to walk tomorrow.* *User: I’ll go out at 7 o’clock.* *Wysa: It’s a nice plan. Shall we record your feelings tomorrow?*	[6,9-18]
Relaxation and mindfulness	Reduced anxiety and stress; improved sleep quality and emotional calmness	Somzz, XiaoE, Wysa	*Somzz: What have you tried to reduce tension before sleep?* *User: I haven’t done much.* *Somzz: Good. Let’s take a deep breath together for 5 minutes. Inhale deeply and slowly exhale.* *(After practice)* *Somzz: Are you a little more comfortable?*	[10,13,14]
Emotional expression and support	Reduction in depression and anxiety; enhanced alliance; and reduced isolation	Woebot, Tess, Wysa, XiaoE	*Tess: What happened today?* *User: I argued with my friend and feel lonely.* *Tess: It’s natural to feel that way. Thank you for telling me.* *Wysa: Can you talk more about the feelings you felt in that*	[6-8,10,12,13]
Self-monitoring and feedback	Reduction in depression and anxiety; and improved self-efficacy	Woebot, Youper, XiaoE, Limbic Care, Wysa	*Woebot: Did you have a moment today when you experienced emotional changes?* *User: I felt bad after I quarreled with my friend this morning.* *Woebot: What if you express your feelings in words?* *User: Anger.* *Woebot: Good. Let’s think about the message that feeling conveys. Perhaps you want to be understood.*	[6,7,9,10,18,19]
Therapeutic alliance formation	Increased user participation, sustainability, symptom relief, and reduced loneliness	Woebot, Tess, Wysa, XiaoE, Therabot	*User: I can’t talk to anyone these days. I feel so lonely.* *Wysa: It’s natural to feel lonely. I understand that feeling.* *Wysa: This space is safe for you to express all your emotions. Let’s get through this together.* *User: Thank you. Just being listened to helps me feel a little better.* *Wysa: I’m always here. You are not alone.*	[6-8,10,12,13,17]

### Cognitive Restructuring

Cognitive restructuring is a central element in CBT, involving the identification and correction of maladaptive thinking. CBT chatbots such as Woebot and XiaoE, Fido, Youper, and Therabot facilitated the identification and reframing of negative thoughts using Socratic questioning. For example, users were prompted with questions such as “Is there evidence for that belief?,” which led them to reframe maladaptive cognitions. Studies confirmed that this process significantly contributed to symptom improvement [6,10]. Mason et al [16] further showed that reductions in cognitive distortions were associated with improvements in depressive symptoms, consistent with mediation, although formal mediation analyses were not conducted. Cognitive distortions were measured using the Cognitive Distortions Scale.

### Behavioral Activation

Another key therapeutic component is behavioral activation, aimed at increasing engagement in goal-directed activities. Woebot encouraged weekly goal setting, whereas Wysa and Fido suggested personalized tasks, and other platforms such as XiaoE, Somzz, Youper, Therabot, Shim, and CBT-TXT also integrated behavioral activation strategies (eg, planning pleasant events, scheduling exercise, or assigning weekly activity tasks). These interventions promoted daily structure, increased activity levels, and reduced avoidance behaviors, contributing to improvements in affect and functional outcomes [6,10,13,15]. Mason et al [16] also found that increases in behavioral activation were associated with improvements in depressive symptoms, in a manner consistent with mediation, although formal mediation analyses were not performed. Behavioral activation was assessed using the Behavioral Activation for Depression Scale.

### Relaxation and Mindfulness

Several CBT chatbots (eg, Somzz, XiaoE, Wysa, and Tess) incorporated structured stress-regulation techniques, including breathing exercises, mindfulness prompts, and grounding strategies. These tools promoted autonomic calming and attentional shift. User feedback emphasized the utility of such exercises for managing anxiety and enhancing emotional calmness [7,8,11]. He et al [10] further reported that the inclusion of mindfulness modules was linked to higher satisfaction and stronger alliance scores.

### Emotional Expression and Support

CBT chatbots provided a psychologically safe environment where users could articulate emotions without judgment. Woebot, Tess, Wysa, and XiaoE all featured empathetic design elements that validated user emotions and reduced feelings of isolation. Wysa users frequently reported feeling emotionally supported, often citing phrases such as “You’re not alone” as impactful [7]. Fulmer et al [8] showed that supportive SMS text messaging design in Tess increased positive affect, and XiaoE trials demonstrated similar benefits through empathic dialogue.

### Self-Monitoring and Feedback

Self-monitoring and feedback were common features in Woebot, Youper, XiaoE, Wysa, and Limbic Care. These platforms incorporated mood check-ins, symptom tracking (eg, PHQ-9 and GAD-7), and personalized feedback loops. Such features helped users recognize symptom patterns and reinforced self-management behaviors. For instance, Youper’s regular mood check-ins contributed to a 3.6-point PHQ-9 reduction within 2 weeks [9], and Wysa data showed that higher engagement was linked to PHQ-9 reductions of over 5 points [7]. The NHS evaluation of Limbic Care also highlighted its role in providing between-session monitoring and feedback in real-world care pathways.

### Development of Therapeutic Alliance

Although chatbots lack human warmth, several studies demonstrated the formation of moderate therapeutic alliance, which promoted sustained engagement and adherence. Alliance was reported or inferred in Woebot, Tess, Wysa, XiaoE, and Therabot. For example, XiaoE users reported higher alliance scores compared to e-book controls [10], whereas Therabot measured alliance using the standardized Working Alliance Inventory–Short Revised and showed moderate levels (mean≈3.6). Wysa users also described a strong sense of rapport, and Woebot/Tess used supportive and empathetic language that fostered trust. Although not equivalent to human-delivered therapy, these alliances contributed to a virtuous cycle of engagement and symptom improvement.

### Comparative Analysis of Technological and Clinical Features of CBT Chatbots

CBT-based mental health chatbots vary widely in their technological design, therapeutic protocols, usability, and patterns of user engagement. A systematic comparison of these characteristics is essential for evaluating their clinical feasibility and technical robustness while also informing the development of next-generation digital mental health interventions. Table 4 presents a comparative summary of key CBT chatbot platforms, including launch year, core AI methods, therapeutic strategies, evidence from empirical studies, and user engagement indicators.

**Table 4.** Comparison of major AI^a^-based mental health chatbots in terms of developer or organization, core AI technologies, implemented CBT^b^ therapeutic protocols, usability, and user engagement. All listed chatbots deliver CBT-based content but differ in conversational style, technical architecture, and adaptivity. “Usability/acceptability” is summarized from user feedback, satisfaction surveys, and system usability scales. “User engagement” reflects indicators such as program completion rates, frequency of use, retention, and dropout. “Partial” indicates limited or indirect implementation of a feature. “NR^c^” denotes information not reported in the study.

Chatbot (developer)	AI technology	Therapeutic protocol						Usability/ acceptability	User engagement
		Cognitive restructuring	Behavioral activation	Relaxation mindfulness	Emotional support	Self-monitoring	Therapeutic alliance		
Woebot (Woebot Health, USA)	NLP^d^, emotion recognition, RNN^e^/transformer-based language model, ML^f^	✓	✓	✓	✓	✓	✓	Very high	Very high
Tess (X2AI/ Cass, USA)	NLP, emotion analysis, rule-based system	**✓**		**✓**	✓	✓	✓	High	Moderate
Wysa (Touchkin, India)	NLP, emotion recognition, ML, affective interaction AI	✓	✓	✓	✓	✓	✓	High	Very high
Somzz (AIMMED, S. Korea)	Rule-based algorithm (CBT-I^g^ focused)	✓	✓	✓	✓	✓		High	Moderate
XiaoE (Tsinghua Univ, China)	NLP with keyword matching+retrieval-based responses	✓	Partial	✓	✓		✓	High	NR
Fido (Univ. of Warsaw, Poland)	Rule based with on-demand chat (hybrid)	✓	✓					NR	Moderate
Youper (Youper Inc, USA)	Custom LLM^h^, emotion analysis, reinforcement learning (hybrid NLP)	✓	✓	Partial	✓	✓	✓	High	Very high
Therabot (Dartmouth/NEJM AI, USA)	Generative AI (LLM, GPT based), emotion recognition	✓	✓	Partial	✓	✓	✓	High	High
Limbic Care (UK, NHS, JMIR)	Generative AI–enabled support tool integrated with therapist-guided CBT	Partial	✓		✓	✓	Partial	High	High

a AI: artificial intelligence.

b CBT: cognitive behavioral therapy.

c NR: not reported.

d NLP: natural language processing.

e RNN: recurrent neural network.

f ML: machine learning.

g CBT-I: cognitive behavioral therapy for insomnia.

h LLM: large language model.

### AI Architecture and Language Design

Early CBT chatbots such as Woebot and Tess were built on rule-based frameworks using decision trees and clinician-authored dialogue scripts. These systems rely on predefined response options triggered by user input, often supported by keyword or sentiment recognition algorithms [6,8]. For example, Woebot used natural language pattern matching to select the most relevant therapist-approved response, ensuring therapeutic consistency and risk minimization. Tess operated similarly, delivering structured interventions via SMS text messaging. While this rule-based approach promotes safety and reliability, it limits scalability and often results in repetitive or nonpersonalized conversations.

Recent developments have introduced more flexible hybrid models. Wysa, for instance, applies sentiment analysis and intent recognition to select responses from a large database, balancing variation with control [7]. Youper combines structured CBT strategies with more advanced NLP, enabling emotionally attuned and dynamic dialogue. To mitigate clinical risks, these hybrid systems still incorporate rule-based safeguards for screening user input and directing users to emergency services when necessary. Youper’s successful deployment, with more than 4500 users retained within 1 month [9], demonstrates the scalability of such hybrid approaches.

### Structure of Therapeutic Protocols

CBT chatbots differ in the structure and flexibility of their therapeutic content. Woebot offers structured, sequential modules (eg, a 2-week program focusing on cognitive restructuring followed by behavioral activation). In contrast, Wysa and Tess provide nonlinear, user-initiated conversations that allow for spontaneous topic switching and indefinite usage duration. Fido represents a hybrid model with an 8-week structured CBT program supplemented by on-demand conversational support between sessions [13].

These structural variations influence research methodology: modular interventions are typically evaluated through RCTs, whereas open-ended chatbots are assessed in real-world observational studies or mixed-method designs [7,9]. Each design has inherent trade-offs—modular programs offer higher experimental control and fidelity, whereas open-ended systems may provide enhanced ecological validity but present challenges for standardization. These findings highlight that the structural format of chatbot protocols interacts meaningfully with both clinical outcomes and user engagement metrics.

### Usability and User Engagement

User-centered design and ease of access are critical to the success of chatbot-based interventions. Across platforms, most CBT chatbots received high usability ratings. For instance, in a postpartum trial, 91% of Woebot users reported satisfaction and 74% completed the full 6-week program [12]. Tess showed near-zero dropout among university students [8]. Longitudinal usage data from Wysa revealed that 33% of users remained active after 2 weeks, and approximately 20% engaged with the chatbot at least twice weekly [7]. These retention figures exceed those of most stand-alone wellness apps and underscore the importance of accessible design and behavioral nudges (eg, reminders and supportive messages).

However, several challenges remain. Users in multiple studies cited limitations such as repetitiveness and reduced emotional depth [6,7]. Some users expressed a preference for more emotionally intelligent and context-aware dialogue. Moreover, while most chatbots include safety protocols to detect crisis keywords (eg, suicidal ideation), these responses are often basic and lack localized nuance or empathic richness. Chatbots operating in linguistically under-resourced contexts, such as Korean, Arabic, or Swahili, face additional challenges due to the limited availability of annotated NLP corpora, which may compromise semantic accuracy and emotional responsiveness. Nonetheless, promising evidence supports their cross-linguistic viability. For example, Shin et al [14] evaluated a Korean-language CBT-I chatbot and found high levels of satisfaction alongside significant reductions in insomnia symptoms. This underscores the importance of ongoing investment in multilingual NLP development to promote global mental health equity.

## Discussion

### Key Findings and Implications

This review of 14 studies indicates that CBT-based chatbots can produce measurable short-term reductions in depressive symptoms, with mixed evidence for anxiety outcomes. These findings align with prior evidence [6,20,21] while also resonating with recent developments in the field of digital mental health. In particular, emerging GenAI chatbots, such as Therabot [17] and Limbic Care [19], have demonstrated substantial clinical benefits and improved engagement, providing complementary evidence that automated conversational agents can be effective in diverse settings. Taken together, these results suggest that CBT-based chatbots may hold promise as adjuncts to mental health care while also underscoring significant challenges that need to be addressed before widespread integration into routine practice.

Consistent with previous research, CBT-informed chatbots generally outperformed passive controls, such as waitlists or psychoeducational apps, in terms of symptom reduction, emotional relief, and user engagement. While results against active comparators (eg, human-guided therapy or coaching apps) were mixed, these tools consistently demonstrated moderate effect sizes for depressive symptoms, underscoring their potential as first-line or supplementary digital interventions.

The therapeutic mechanisms observed across studies closely mirrored the foundational components of CBT, including cognitive restructuring [16], behavioral activation, mindfulness-based stress regulation, and simulated empathic interaction [8,10]. These features were delivered through structured, interactive dialogue that enabled users to challenge maladaptive cognitions, build adaptive behavioral routines, and receive emotionally validating responses. Many chatbots implemented a CBT-consistent therapeutic loop in which cognitive change facilitated symptom relief and behavioral re-engagement—a digital analog of the traditional CBT model [21]. Conceptually, such systems can be described as “cognitive-affective artifacts”—designed tools that modulate cognitive and affective states to support therapeutic processes.

In addition to clinical effectiveness, CBT chatbots offer advantages in scalability, anonymity, and 24/7 accessibility. Their use has been successfully demonstrated in diverse populations, from college students to postpartum women [8,12]. These platforms may serve not only as symptom management tools but also as low-threshold entry points for psychological support—functioning as digital companions during acute distress or between therapy sessions [7].

Nevertheless, several challenges remain. Although no study reported serious adverse events, most samples involved individuals with mild-to-moderate symptoms, raising concerns about applicability in more severe cases. For individuals with severe depression, psychosis, or suicidal ideation, chatbots should be integrated into a broader care plan with robust crisis intervention protocols [22]. Variability in study designs, comparator conditions, outcome measures, and chatbot platforms further complicates direct comparisons, a challenge also noted in recent reviews such as Zhong et al [20]. Standardized evaluation frameworks are needed to address this heterogeneity.

### Future Research Priorities

First, longitudinal studies are essential to determine whether short-term benefits persist over time. Some findings suggest that gains diminish after the intervention period [13,22], whereas others indicate sustained effects when usage is extended or supplemented with booster sessions. Second, adaptive machine learning could enhance personalization by tailoring content, pacing, and dialogue dynamically to user characteristics, thereby improving alignment and retention. Third, the integration of generative large language models such as ChatGPT or GPT-4 may deepen conversational empathy and alliance but must be paired with rule-based safeguards and clinical oversight to prevent misinformation or ethical risks [21]. Finally, broader policy and implementation issues require attention, including regulatory oversight (eg, FDA SaMD frameworks), privacy and data security (Health Insurance Portability and Accountability Act/General Data Protection Regulation compliance), and financial sustainability models to support scalable integration into health care systems.

Attention should also be directed to linguistic and cultural inclusivity. Encouraging results from non-English platforms, such as the Korean-language CBT-I chatbot studied by Shin et al [14], demonstrate that CBT-based interventions can be adapted effectively to diverse sociocultural contexts. Investment in multilingual NLP and culturally sensitive design will be critical for equitable global dissemination.

### Limitations

This review has several limitations. First, the evidence base remains limited in scope and quality: many studies involved small samples, short intervention periods, and heterogeneous outcome reporting, with moderate risk of bias in several cases. Moreover, most included chatbots were rule-based systems, with only a few recent platforms (eg, Therabot and Limbic Care) incorporating generative AI, constraining generalizability. Second, as a narrative review, this synthesis is inherently subject to selection bias, interpretive subjectivity, and potential publication bias, despite the use of predefined inclusion criteria and dual-reviewer procedures. Future research should examine whether emerging generative AI chatbots differ in efficacy, engagement, and safety compared with traditional rule-based designs.

### Conclusions

CBT-based chatbots represent a clinically grounded, scalable, and contextually adaptable modality for mental health care delivery. They hold particular promise for increasing access to evidence-based support in underserved populations. As this technology continues to mature, future efforts should emphasize long-term efficacy, adaptive personalization, clinician-guided integration, and rigorous ethical oversight. Recent advances in generative AI chatbots (eg, Therabot and Limbic Care) further highlight both opportunities and challenges for implementation. Rather than replacing human therapists, chatbot systems are best positioned to augment existing services and bridge care gaps in increasingly strained global mental health systems.

## Abbreviations

AI: artificial intelligence
CBT: cognitive behavioral therapy
CBT-I: cognitive behavioral therapy for insomnia
GAD-7: Generalized Anxiety Disorder-7
GenAI: generative AI
NLP: natural language processing
PHQ-9: Patient Health Questionnaire-9
RCT: randomized controlled trial
RoB 2.0: Risk of Bias 2.0
